# The effect of chronic yoga interventions on sleep quality in people with sleep disorders: a scoping review

**DOI:** 10.3389/fneur.2025.1566445

**Published:** 2025-04-29

**Authors:** Mohammad Alghosi, Maryam Sharifi, Sanam Namavari, Neda Rajamand, Faezeh Bamorovat, Narges Norouzi, Mohammad Alimoradi, Andreas Konrad

**Affiliations:** ^1^Department of Sports Injury and Corrective Exercise, Faculty of Physical Education and Sport Sciences, University of Guilan, Rasht, Iran; ^2^Department of Physical Education and Sport Science, National University of Skill (NUS), Tehran, Iran; ^3^Department of Sports Injuries and Corrective Exercises, Faculty of Sports Sciences, Shahid Bahonar University of Kerman, Kerman, Iran; ^4^Department of Health and Sport Medicine, Faculty of Physical Education and Sport Sciences, University of Tehran, Tehran, Iran; ^5^Department of Exercise Physiology, Faculty of Physical Education and Sport Sciences, Kharazmi University, Tehran, Iran; ^6^Faculty of Sport Sciences, Alzahra University, Tehran, Iran; ^7^Institute of Human Movement Science, Sport and Health, Graz University, Graz, Austria

**Keywords:** sleep problem syndrome, yoga, sleep quality, exercise therapy, non-pharmacological treatment

## Abstract

**Background:**

Poor or insufficient sleep adversely affects various physiological and psychological functions, impacting body systems such as the endocrine, metabolic, and immune systems.

**Objectives:**

Despite available pharmacological and non-pharmacological treatments, the impact of chronic yoga interventions on sleep quality in individuals with sleep problem syndrome remains underexplored. This scoping review aims to consolidate existing research on yoga interventions and their effects on sleep quality, providing evidence for yoga as a non-pharmacological alternative to reduce reliance on medications.

**Methods:**

A systematic search across PubMed, Web of Science, and Scopus identified 1,559 studies, with 57 meeting inclusion criteria for yoga’s effects on sleep quality.

**Results:**

Overall, the included studies reported either significant improvements in sleep quality (or related) parameters or no change. Moderator analyses revealed that intervention duration and session frequency can influence sleep outcomes. Short-duration interventions (≤6 weeks) showed a large mean effect on sleep quality (9.41%; 95% CI 3.06 to 15.42%), with 54% of studies reporting statistically significant improvements. Medium-duration interventions (7–16 weeks) demonstrated consistent benefits, including a large mean effect on sleep quality (8.74%; 95% CI 2.93 to 14.55%) and a very large reduction in insomnia severity (13.19%; 95% CI 11.10 to 15.98%). However, sleep efficiency exhibited smaller effects (0.73%; 95% CI −1.99 to 3.45%). Long-duration interventions (≥17 weeks) produced the most robust results, with 100% of the studies reporting significant improvements, including a 7.92% increase in sleep quality (95% CI 3.23 to 12.60%). With regard to session frequency, low-frequency sessions (1–2 per week) yielded significant improvements in insomnia severity (13.66%; 95% CI 8.72 to 18.59%) and sleep quality (8.13%; 95% CI 2.67 to 13.59%). Moderate-frequency sessions (3–4 per week) balanced accessibility and efficacy, producing a large mean effect on sleep quality (9.21%; 95% CI 3.66 to 14.76%). High-frequency sessions (≥5 per week) demonstrated a similarly large effect on sleep quality (8.24%; 95% CI 2.28 to 14.20%), although the data were limited.

**Conclusion:**

Tailoring yoga interventions by duration and frequency is valuable, with chronic practice offering a safe, effective alternative to medication. Future research should refine protocols for specific populations and sleep challenges.

## Highlights

- The scoping review highlights that chronic yoga practice significantly improves sleep quality in individuals with sleep problem syndrome. Short, medium, and long-duration yoga interventions were shown to produce varying degrees of improvement in sleep parameters, with long-duration interventions yielding the most robust results.
- The effectiveness of yoga in enhancing sleep quality is influenced by the duration of the intervention and the frequency of sessions. Short-duration interventions (≤6 weeks) and low-frequency sessions (1–2 times per week) demonstrated significant improvements in insomnia severity and sleep quality, while longer interventions (≥17 weeks) consistently led to substantial positive changes.
- The findings support the use of yoga as a safe and effective non-pharmacological alternative to pharmacological treatments for sleep disturbances. This approach may reduce reliance on medications and address sleep problems holistically, emphasizing the importance of tailored yoga protocols based on individual needs.

## 1 Introduction

Sleep problems are among the most frequent medical complaints encountered in clinical practice (1). Insufficient sleep is linked to notably reduced work performance, impaired daytime functioning, and higher health care costs (2, 3). Poor or insufficient sleep affects a variety of physiological and psychological functions, impacting multiple body systems, including the endocrine, metabolic, and immune systems. This compromises higher cortical functions, cognitive performance, mood, and recovery after physical activity (4, 5). Sleep disturbances can affect both the duration and quality of sleep, leading to significant reductions in functionality and overall quality of life (6).

In the United States, the economic burden of diagnosing and treating sleep-related issues, including medical treatments, reached approximately $12.4 billion in 2015 (7). About 30% of the general population suffers from sleep disorders, with 10% experiencing disrupted sleep patterns and daytime dysfunction (8). Research indicates that the prevalence of sleep disorders ranges from 9 to 12% in adults and increases to 20% to 30% in older adults, highlighting the growing public health concern (9, 10). The management of sleep disorders currently involves both pharmacological and non-pharmacological approaches. While pharmacotherapy is commonly used, it is generally only recommended for short-term use, due to potential risks such as hazardous side effects, tolerance, and dependency associated with long-term use (11, 12).

Non-pharmacological interventions aimed at improving sleep encompass a variety of strategies, including sleep hygiene (13, 14), stimulus control (15, 16), muscle relaxation therapy (16–18), sleep restriction therapy (19, 20), and cognitive therapy (20) for insomnia. In addition, bright light therapy (21, 22) and exercise (23–31) are also considered effective methods (31) for enhancing sleep quality. These approaches focus on changing poor sleep habits and addressing negative thoughts, attitudes, and beliefs about sleep.

Among the non-pharmacological treatments related to exercise, yoga has been extensively adopted in various forms across both the Eastern and Western hemispheres. This ancient practice emphasizes strength, flexibility, and breathing to enhance physical, mental, and spiritual well-being (30). There are numerous styles of yoga, including Tibetan, Iyengar, and hatha yoga, each with its unique focus and intensity. Some styles are more physically demanding, while others concentrate on different aspects, such as posture (asanas) or breathing (pranayama) (30). In Europe and America, yoga practice typically centers on these primary components, along with meditation (dhyana), to promote overall well-being.

Numerous studies have shown that yoga is a safe and effective intervention for improving fatigue severity, depressive moods, and sleep quality in various populations (32, 33). Yoga is also considered a mindful form of physical activity. Mindfulness, a key element of yoga, helps improve sleep disturbances by increasing melatonin levels, reducing hyperarousal, and addressing stress-related cardiac and respiratory abnormalities (30). However, some research has reported limited or no significant effects of yoga on sleep quality, suggesting that the benefits may not be universal across all populations or conditions (34, 35). The integration of mindfulness in yoga practice can lead to improved sleep quality and duration, offering a holistic approach to managing sleep disorders (36). Therefore, despite the growing body of evidence supporting yoga’s effects on sleep quality, it is essential to summarize the findings in a comprehensive review, to evaluate its effectiveness in addressing sleep problems across different populations.

This scoping review aims to synthesize current research on the impact of chronic yoga practice on the various parameters that assess sleep quality (e.g., sleep duration, sleep efficiency, sleep disturbances, etc.), identify gaps in the literature, and provide recommendations for future studies.

## 2 Methods

The authors conducted a scoping review due to the high variability among the included studies in terms of sleep quality parameters and the FITT (Frequency, Intensity, Time, and Type) in yoga interventions, in order to provide an overview of the existing literature on this topic. This variability necessitated a scoping review approach, as recommended by Munn et al. (37) regarding the appropriate methodology for this type of literature review. The authors referred to the Preferred Reporting Items for Systematic Reviews and Meta-Analyses (PRISMA) Scoping Review Checklist to ensure quality and adequacy of reporting, as outlined by Tricco et al. (38).

### 2.1 Information sources and search strategy

A comprehensive literature search was conducted independently by two authors (F.B. and N.N.) across the PubMed, Web of Science, and Scopus databases, from inception to July 10, 2024. The key search terms used included:

- *Sleep-related terms*: “sleep problem,” “sleep disorder,” “sleep complaints,” “sleep disturbance,” “sleep quality,” “dyssomnia,” “extrinsic sleep disorder,” “sleep initiation and maintenance disorder.”
- *Yoga-related terms*: “yoga.”

The search was conducted using a Boolean strategy with the OR operator to combine these terms [e.g., (“sleep problem” OR “sleep disorder” OR “sleep complaints” OR “sleep disturbance” OR “sleep quality” OR “dyssomnia” OR “extrinsic sleep disorder” OR “sleep initiation” and “maintenance disorder”) AND “yoga”]. The literature search was restricted to full-text articles published in English, German, or Persian. In addition, a supplementary search was conducted by reviewing the reference lists of identified original and review articles, and through the Connected Papers website^1^ to find other relevant studies.

### 2.2 Eligibility criteria

The inclusion and exclusion criteria for the studies were based on the PICOS (Population, Intervention, Comparison, Outcome, and Study design) framework (39), as outlined in Table 1.

**Table 1:** Eligibility of the studies based on the PICOS framework.

	Inclusion criteria	Exclusion criteria
Population	Individuals with sleep problems Any sex	Participants using pharmacological treatments
Intervention	Studies that investigated the effects of yoga as the only intervention on sleep quality	Studies where yoga was used alongside other interventions or techniques
Comparison	Studies that provided pre-to-post only (no control) comparisons or included a control condition	NA
Outcomes	Studies with sleep quality measures	NA
Study type	Published articles up until June 2024 Written in English, German, or Persian	Publications without full text Academic theses, books, or non-scientific articles

### 2.3 Study selection

**Figure 1:**
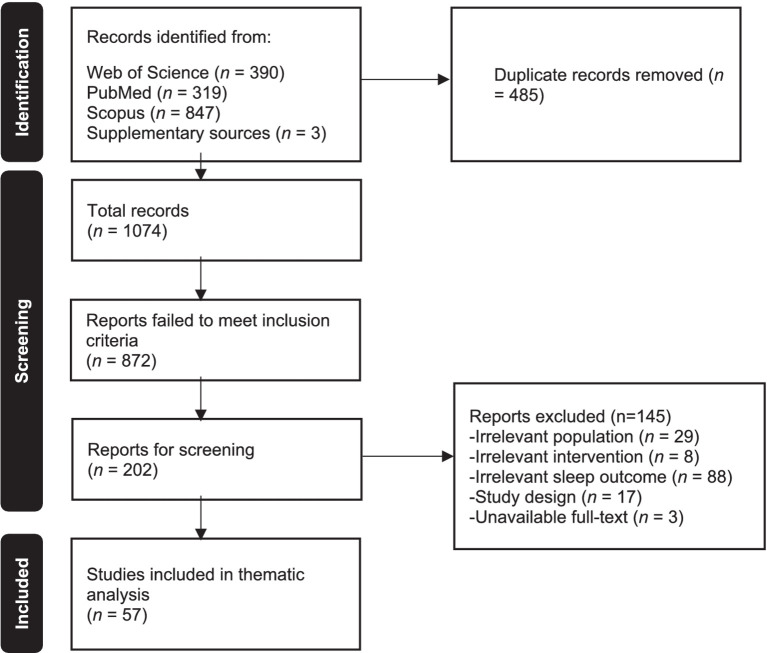
PRISMA 2020 flow diagram.

The study selection process involved two independent reviewers (M.ALI. and M.ALG.), who screened the titles, abstracts, and full-text articles based on predefined inclusion and exclusion criteria. The process was carried out in two stages. In the first stage, the reviewers assessed the titles and abstracts of the identified articles. Articles that appeared relevant were moved to the next stage, while those that were clearly irrelevant were excluded. In the second stage, the full-text articles of potentially relevant studies were assessed in detail to determine eligibility. Any disagreements between the reviewers during this process were resolved through discussion, and if necessary, a third reviewer (N.R.) was consulted to make the final decision on eligibility. The number of studies screened, excluded, and included at each stage of the selection process is depicted in a PRISMA 2020 flow diagram (Figure 1).

### 2.4 Data extraction

Two independent reviewers (F.B. and N.N.) extracted data using a standardized spreadsheet, capturing study details (authors, year, design), participant characteristics (sample size, age, sex, medical conditions), yoga intervention specifics (type, frequency, duration), and sleep-related outcomes (e.g., latency, efficiency, disturbance). For controlled studies, between-group differences were recorded. To ensure accuracy, both reviewers cross-checked their extractions against the original articles and resolved discrepancies through discussion, with unresolved cases adjudicated by a third reviewer (N.R.). Prior to full extraction, a pilot test on three studies confirmed consistency in methodology.

### 2.5 Synthesis of results

A thematic analysis approach was employed to identify key themes and patterns across the included studies. The findings were summarized narratively, and tables or diagrams were used to present the results. The main outcome measures included sleep quality parameters such us sleep latency, sleep duration, sleep efficiency, sleep disturbance, sleep medication, and daytime dysfunction. In addition, moderating variables, as in the following, were considered in synthesizing the findings, including intervention duration, session frequency per week, types of yoga intervention, and population type. The following sections outline the percentage-weighted mean changes (from pre- to post-intervention), along with the corresponding 95% confidence intervals (CIs), highlighting the impact of yoga interventions on sleep outcomes. In accordance with prior recommendations, we classified the calculated percentage-weighted mean changes in the parameters into distinct magnitudes: changes under 0.5% were deemed trivial, those between 0.5% and less than 2% were categorized as small, 2% to less than 5% as moderate, 5% to less than 10% as large, and changes exceeding 10% as very large (40, 41).

## 3 Results

### 3.1 Search results

Initially, a total of 1,559 records were identified through both the electronic database searches and manual reviews, which included searching citation lists to identify additional relevant studies. After removing duplicates, 1,074 publications advanced to the title and abstract screening stage. From this pool, 202 studies underwent a full-text assessment, where 145 studies were excluded for various reasons (see Figure 1). Finally, a total of 57 publications were included for the thematic analysis, concentrating on the effects of chronic yoga practice on sleep quality.

### 3.2 Characteristics of the included studies

The articles included in this study were published between 2004 and 2024. The overall sample size across all studies was 6,057 participants; however, the population size varied among the articles, ranging from *n* = 13 to *n* = 820. The average age of participants ranged from 15 ± 1.50 to 75.40 ± 6.70 years. In terms of sex distribution, a total of 4,856 participants (80.04%) were female, while 1,169 (19.27%) were male. In addition, the sex of 41 participants (0.67%) was not reported, while one participant (0.02%) chose not to reveal their sex. Among the 57 studies analyzed, 40 were randomized controlled trials (RCTs), 4 were non-RCTs (i.e., controlled trials), and 13 were non-controlled studies. The results are presented in Table 2.

**Table 2:** Characteristics of the included studies.

Study details	Study design	Participants	Yoga characteristics	Main outcome measured	Percentage change (pre to post) of the main outcome	Difference to control (intervention minus control)
1. Elavsky and McAuley, 2007 (93)	RCT	*n*: 164 Age: 49.90 ± 3.60 Sex: F Medical condition: healthy	Type: Iyengar yoga Frequency (times per week): 2 Time (min): 90 Duration (weeks): 16	*(PSQI)*		
				Total score	↓ 6.69%^NR^	1.24 %^NS^
				Sleep quality	↑ 1.20% ^NR^	0.16 %^NS^
				Sleep latency	↓ 0.93% ^NR^	0.14 %^NS^
				Sleep duration	↓ 0.88% ^NR^	0.20 %^NS^
				Habitual sleep efficiency	↑ 0.51% ^NR^	0.10 %^NS^
				Sleep disturbance	↓ 1.50% ^NR^	0.23 %^NS^
				Use of sleep medication	↑ 0.58% ^NR^	0.25 %^NS^
				Daytime dysfunction	↑ 1.03% ^NR^	0.11 %^NS^
2. Carson et al., 2009 (85)	RCT	*n*: 37 Age: 54.40 ± 7.50 Sex: F Medical condition: breast cancer survivors	Type: Yoga of Awareness Frequency (times per week): 1 Time (min): 120 Duration (weeks): 8	Sleep disturbance	↓ 3.55%^NR^	1.36%^S^
3. Danhauer et al., 2009 (94)	RCT	*n*: 44 Age: 55.75 ± 9.90 Sex: F Medical condition: breast cancer	Type: restorative yoga Frequency (times per week): 1 Time (min): 75 Duration (weeks): 10	PSQI score	↓ 7.48%^NR^	−2.11%^NS^
4. Chandwani et al., 2010 (95)	RCT	*n*: 61 Age: 50.18 ± 8.98 Sex: F Medical condition: breast cancer	Type: Patanjali yoga Frequency (times per week): 3 Time (min): 60 Duration (weeks): 6	PSQI score	↔	↔
5. Chen et al., 2010 (42)	RCT	*n*: 55 Age: 75.40 ± 6.70 Sex: F = 29, M = 26 Medical condition: depression	Type: silver yoga Frequency (times per week): 3 Time (min): 70 Duration (weeks): 24	*(PSQI)*		
				Total score	↓ 4.24%^S^	−1.80% ^S^
				Sleep duration	↑ 0.61%^NS^	−0.20% ^S^
				Habitual sleep efficiency	↑ 0.53%^NS^	−0.23% ^S^
				Sleep disturbance	↓ 0.61%^S^	−0.06% ^S^
				Daytime dysfunction	↓ 0.16%^S^	−0.13% ^S^
6. Afonso et al., 2012 (76)	RCT	*n*: 44 Age: 50–65^‡^ Sex: F Medical condition: insomnia	Type: yogasana and Tibetan Frequency (times per week): 2 Time (min): 60 Duration (weeks): 16	Insomnia Severity Index	↓ 11.91%^S^	−2.55%^S^
7. Bower et al., 2012 (66)	RCT	*n*: 31 Age: 53.86 ± 5.31 Sex: F Medical condition: breast cancer survivors	Type: Iyengar yoga Frequency (times per week): 2 Time (min): 90 Duration (weeks): 12	PSQI score	↓ 8.65%^NS^	−0.25%^NS^
8. Innes and Selfe, 2012 (43)	RCT	*n*: 20 Age: 58.40 ± 2 Sex: F Medical condition: RLS	Type: Iyengar yoga Frequency (times per week): 2 Time (min): 90 Duration (weeks): 8	Average sleep duration in hours	↑ 6.52%^S^	−0.18% ^S^
				Prevalence of insomnia	↓ 75%^S^	45%^S^
				*(PSQI)*		
				Global score	↓ 6.14%^S^	−2.48% ^S^
				Sleep latency	↔	−1.37%^NS^
				Sleep quality	↑ 1.06%^NS^	−0.56%^S^
				Sleep duration	↑ 1%^S^	−0.35% ^S^
				Sleep efficiency	↑ 1.19%^S^	0.62% ^S^
				Sleep disturbance	↓ 1.42%^S^	−0.32% ^S^
				Sleep medication	↓ 0.06%^NS^	−0.69%^NS^
				Daytime dysfunction	↓ 1.44%^S^	0.01% ^S^
9. Kohn et al., 2013 (96)	RCT	*n*: 37 Age: 53.02 ± 11.91 Sex: F = 34, M = 3 Medical condition: stress	Type: medical yoga Frequency (times per week): 1 Time (min): 60 Duration (weeks): 12	Insomnia Severity Index	↓ 9.70%^NR^	−5.20%^NS^
10. Mustian et al., 2013 (44)	RCT	*n*: 410 Age: 54.01 ± 0.51 Sex: *F* = 393, M = 17 Medical condition: insomnia	Type: hatha and restorative yoga Frequency (times per week): 2 Time (min): 70 Duration (weeks): 4	*(PSQI)*		
				Global score	↓ 8.21%^S^	0.54%^NS^
				Sleep latency	↓ 1.31%^S^	−0.06% ^NS^
				Sleep duration	↑ 0.96%^S^	0.01% ^NS^
				Sleep efficiency	↑ 0.94%^S^	−0.02% ^NS^
				Sleep disturbance	↓ 1.58%^S^	−0.08% ^NS^
				Daytime dysfunction	↓ 1.11%^S^	−0.02%^S^
				Sleep medication use	↓ 0.90%^NS^	0.08% ^S^
				Sleep quality	↑ 1.39%^S^	−0.10% ^S^
				*(Actigraphy)*		
				Sleep onset latency	↓ 0.49%^NS^	−0.05% ^NS^
				Wake after sleep onset	↓ 1.03%^NS^	−0.06% ^NS^
				Overall sleep efficiency (%)	↑ 0.76%^NS^	−0.70%^S^
11. Hariprasad et al., 2013 (97)	RCT	*n*: 120 Age: 75.27 ± 5.93 Sex: *F* = 72, M = 48 Medical condition: healthy	Type: integrated yoga Frequency (times per week): 7 Time (min): 60 Duration (weeks): 12	PSQI score	↓ 7.10% ^NR^	−1.22%^S^
12. Chandwani et al., 2014 (98)	RCT	*n*: 163 Age: 51.86 ± 1.33 Sex: F Medical condition: breast cancer	Type: integrated yoga Frequency (times per week): 1 Time (min): 60 Duration (weeks): 6	PSQI score	↓ 7.53%^NR^	−0.38%^NS^
13. Cheung et al., 2014 (99)	RCT	*n*: 36 Age: 71.90 ± NA Sex: F Medical condition: osteoarthritis	Type: hatha yoga Frequency (times per week): 1 Time (min): 60 Duration (weeks): 8	*(PSQI)*		
				Total score	↓ 5.75%^NR^	0% ^NS^
				Sleep quality	↑ 0.88% ^NR^	−0.01% ^NS^
				Sleep latency	↓ 1.15% ^NR^	0.24% ^NS^
				Sleep duration	↑ 0.30% ^NR^	0.15% ^NS^
				Sleep disturbance	↓ 1.55% ^NR^	0.10% ^NS^
				Use of sleep medication	↑ 0.68% ^NR^	−0.02% ^NS^
				Sleep efficiency	↑ 0.53% ^NR^	−0.10% ^NS^
14. Kiecolt-Glaser et al., 2014 (87)	RCT	*n*: 200 Age: 51.60 ± 9.20 Sex: F Medical condition: breast cancer survivors	Type: NR Frequency (times per week): 2 Time (min): 90 Duration (weeks): 12	PSQI score	NR	NR%^S^
15. Jindani et al., 2015 (100)	RCT	*n*: 80 Age: 41.00 ± NA Sex: F Medical condition: posttraumatic stress	Type: Kundalini yoga Frequency (times per week): 1 for the group training and 7 for the home Time (min): 90 for the group training and 15 for the home Duration (weeks): 8	Insomnia Severity Index	↓ 12.5%^NR^	−3.75%^S^
16. Fang and Li, 2015 (101)	RCT	*n*: 105 Age: 35.57 ± 10.46 Sex: F Medical condition: healthy	Type: NR Frequency (times per week): 2 Time (min): 50–60 Duration (weeks): 24	*(PSQI)*		
				Sleep quality	↑ 1.54% ^NR^	−0.15% ^S^
				Sleep duration	↑ 1.56% ^NR^	−0.13% ^S^
				Sleep efficiency	↑ 1.58% ^NR^	−0.21% ^S^
				Sleep disturbance	↓ 1.67% ^NR^	−0.24% ^S^
				Use of sleep medication	↓1.60% ^NR^	−0.20% ^S^
				Daytime dysfunction	↓ 1.56% ^NR^	−0.27% ^S^
				Total score	↓ 8.79% ^NR^	−1.48% ^S^
17. Ratcliff et al., 2016 (89)	RCT	*n*: 163 Age: 51.86 ± 1.33 Sex: F Medical condition: breast cancer	Type: NR Frequency (times per week): 1 Time (min): 90 Duration (weeks): 10	PSQI score	↓ 7.50%^NR^	−0.25%^NS^
18. Cramer et al., 2016 (102)	RCT	*n*: 54 Age: 68.30 ± 9.70 Sex: *F* = 21, M = 33 Medical condition: colorectal cancer	Type: NR Frequency (times per week): 1 Time (min): 90 Duration (weeks): 10	PSQI score	↓ 9.05%^NR^	−1.17%^S^
19. Ebrahimi et al., 2017 (45)	RCT	*n*: 39 Age: 46.85 ± 3.35 Sex: F Medical condition: type 2 diabetes	Type: integrated yoga Frequency (times per week): 3 Time (min): 90 Duration (weeks): 12	*(PSQI)*		
				Sleep quality	↑ 1.20% ^S^	−0.43%^NR^
				Sleep latency	↓ 1.30% ^S^	−0.65%^NR^
				Sleep duration	↑ 1.23% ^S^	−0.54% ^NR^
				Sleep efficiency	↑ 1.63% ^S^	−0.46% ^NR^
				Sleep disturbance	↓ 1.63% ^S^	−0.91% ^NR^
				Use of sleeping medication	↓ 1.07% ^S^	−0.93% ^NR^
				Daytime dysfunction	↓ 1.06% ^S^	−0.52% ^NR^
				Total score	↓ 9.06% ^S^	−4.52% ^NR^
20. Buchanan et al., 2017 (103)	RCT	*n*: 186 Age: 54.07 ± 3.69 Sex: F Medical condition: hot flushes	Type: viniyoga yoga Frequency (times per week): 1 for the class sessions and 7 for the home Time (min): 90 for the first session, 75 for the following sessions, and 30 for the home Duration (weeks): 12	*(Actigraphy)*		
				Total sleep time	↑ 6.74 %^NR^	−0.12 %^NS^
				Wake after sleep onset	↓ 0.86% ^NR^	−0.08% ^NS^
				Long awakenings	↓ 2.25% ^NR^	−0.30% ^NS^
				Sleep onset latency	↓ 0.21% ^NR^	−0.05% ^NS^
				Sleep efficiency (%)	↑ 0.85% ^NR^	0.55% ^NS^
21. Ward et al., 2018 (104)	RCT	*n*: 26 Age: 54.00 ± 11.00 Sex: *F* = 25, M = 1 Medical condition: rheumatoid arthritis	Type: viniyoga yoga Frequency (times per week): 1 for the first week, 6 for the next 8 weeks, and NR for the final 4 weeks Time (min): 90 for the first week, 75 for the next 8 weeks, and 30 for the final 4 weeks Duration (weeks): 13	Insomnia Severity Index	↓ 10.50%^NR^	0.90%^S^
22. Nalgirkar et al., 2018 (46)	RCT	*n*: 30 Age: 29.85 ± 4.45 Sex: F Medical condition: dysfunctional uterine bleeding	Type: integrated yoga Frequency (times per week): 3 Time (min): 60 Duration (weeks): 12	*(PSQI)*		
				Sleep quality	↑ 0.83%^NS^	−0.16% ^NS^
				Sleep latency	↓ 1.24%^NS^	0.29% ^NS^
				Sleep duration	↓ 0.87%^NS^	0.50% ^NS^
				Habitual sleep efficiency	↑ 0.36%^NS^	0.07% ^NS^
				Sleep disturbance	↓ 6.87%^S^	−0.17%^S^
				Use of sleep medication	↓ 1.04%^S^	0.29%^S^
				Daytime dysfunction	↓ 1.29%^NS^	0.29%^NS^
				Total score	↓ 13.95%^S^	3.95%^S^
23. Chaoul et al., 2018 (105)	RCT	*n*: 227 Age: 49.58 ± 10.06 Sex: F Medical condition: breast cancer	Type: Tibetan yoga Frequency (times per week): 4 Time (min): 75–90 Duration (weeks): 12	*(PSQI)*		
				Total score	↓ 7.55%^NR^	−0.55%^NS^
				Sleep efficiency	↔	−0.70%^NS^
				Sleep quality	↑ 3.45%^NR^	−0.10%^NS^
				Sleep disturbance	↓ 2.65%^NR^	−0.15%^S^
				*(Actigraphy)*		
				Sleep efficiency (%)	↑ 0.81%^NR^	0.15% ^NS^
				Sleep onset latency	↑ 0.57%^NR^	0.05% ^NS^
				Total sleep time	↑ 7.25%^NR^	0.05% ^NS^
				Wake after sleep onset	↑ 0.74%^NR^	−0.07%^S^
24. Lin et al., 2019 (106)	RCT	*n*: 358 Age: 54.30 ± 10.20 Sex: *F* = 344, M = 14 Medical condition: cancer	Type: hatha and restorative yoga Frequency (times per week): 2 Time (min): 75 Duration (weeks): 4	*(PSQI)*		
				Sleep quality	↑ NR%^NR^	NR%^NS^
				Sleep medication use	↓ NR%^NR^	NR%^NS^
				Daytime dysfunction	↓ NR%^NR^	NR%^S^
				Overall score	↓ NR%^NR^	NR%^S^
25. Huberty et al., 2020 (88)	RCT	*n*: 90 Age: NR Sex: F Medical condition: posttraumatic stress	Type: hatha yoga Frequency (times per week): NR Time (min): NR Duration (weeks): 12	PSQI score	Low dose: ↓ 9.59%^NR^	0.95%^NS^
					Moderate dose: ↓ 7.63%^NR^	1%^NS^
26. Innes et al., 2020 (47)	RCT	*n*: 41 Age: 50.90 ± 2.40 Sex: *F* = 32, M = 9 Medical condition: RLS	Type: Iyengar yoga Frequency (times per week): 2 for gym and 6 home Time (min): 75 for gym and 30 for home Duration (weeks): 12	IRLS score total	↓ 19.06%^S^	No CG
				IRLS impact scale	↓ 4.15%^S^	
				IRLS severity scale	↓ 12.48%^S^	
				RLS severity	↓ 3.73%^S^	
				PSQI score	↓ 10.59%^S^	
27. Datta et al., 2021 (48)	RCT	*n*: 41 Age: 43.29 ± 11.53 Sex: NR Medical condition: insomnia	Type: yoga nidra Frequency (times per week): NR Time (min): NR Duration (weeks): NR	Time in bed	↑ 7.50%^NS^	No CG
				Total sleep time	↑ 5.84%^S^	
				Sleep efficiency (%)	↑ 0.79%^S^	
				Sleep onset latency	↓ 1.24%^S^	
				Wake after sleep onset	↓ 0.46%^S^	
				Total wake duration	↓ 1.65%^S^	
				Sleep quality	↑ 5.41%^S^	
28. Ganesh et al., 2021 (49)	RCT	*n*: 96 Age: 62.60 ± 3.90 Sex: *F* = 60, M = 36 Medical condition: healthy	Type: integrated yoga Frequency (times per week): 3 Time (min): NR Duration (weeks): 12	*(PSQI)*		
				Sleep latency	↓ 0.86%^S^	−0.28% ^S^
				Sleep disturbance	↓ 0.89%^S^	−0.12% ^S^
				Sleep medicine score	↓ 0.03%^NS^	−0.07% ^S^
				Daytime dysfunction	↓ 0.48%^S^	−0.14% ^S^
				Sleep total	↓ 11.72%^S^	−3.28% ^S^
				Sleep duration	↑ 0.91%^S^	−0.27% ^S^
				Habitual sleep efficiency	↑ 0.11%^S^	−0.10% ^S^
29. Khalsa and Goldstein, 2021 (50)	RCT	*n*: 44 Age: 25–59^‡^ Sex: *F* = 23, M = 21 Medical condition: insomnia	Type: Kundalini yoga Frequency (times per week): 7 Time (min): 45 Duration (weeks): 8	Sleep onset latency	↓ 0.63%^S^	No CG
				Awakenings	↓ 1.35%^NS^	
				Wake after sleep onset	↓ 0.39%^S^	
				Total wake time	↓ 1.46%^S^	
				Total sleep time	↑ 6.79%^S^	
				Sleep efficiency (%)	↑ 0.82%^S^	
				Sleep quality	↑ 4.95%^S^	
				Restedness	↑ 4.85%^S^	
				Insomnia Severity Index	↓ 14.10%^NS^	
				Insomnia symptom questionnaire	↓ 43.30%^NS^	
				PSQI score	↓ 10.15%^S^	
				Self-efficacy for sleep	↑ 49.90%^S^	
				PSAS somatic	↓ 12.40%^NS^	
				PSAS cognitive	↓ 13.35%^NS^	
30. Susanti et al., 2022 (51)	RCT	*n*: 208 Age: 52.48 ± 4.06 Sex: F Medical condition: healthy	Type: NR Frequency (times per week): 3 Time (min): 75 Duration (weeks): 20	PSQI score	↓ 7.15%^S^	−5.22%^S^
31. Currie et al., 2022 (77)	RCT	*n*: 445 Age: 18–25^‡^ (6.46%), 26–36^‡^ (29.25%), 37–47^‡^ (20.41%), 48–58^‡^ (18.03%), 59–69^‡^ (24.49%), 70–80^‡^ (1.36%) Sex: *F* = 291, M = 154 Medical condition: insomnia	Type: Yoga of Immortals Frequency (times per week): 7 Time (min): 30 Duration (weeks): 12	*(Insomnia Severity Index)*		
				Severe insomnia	↓ 100%^S¥^	65.2% ^S¥^
				Mild insomnia	↓ 71.43%^S¥^	67.43% ^S¥^
				Moderate insomnia	↓ 68.42%^S¥^	62.36% ^S¥^
32. Eyuboglu et al., 2023 (52)	RCT	*n*: 44 Age: NR Sex: *F* = 4, M = 40 Medical condition: sleep apnea	Type: tele yoga Frequency (times per week): 3 Time (min): 60 Duration (weeks): 12	*(PSQI)*		
				Sleep duration (min)	↑ 6.45%^S^	0.06%^S^
				Sleep efficiency (%)	↑ 0.74%^S^	0.14%^S^
				Sleep duration (score)	↓ 1.20%^NS^	−0.57% ^NS^
				Sleep disturbance	↓ 1.54%^S^	−0.10% ^NS^
				Sleep latency	↓ 1.09%^NS^	−0.18% ^NS^
				Daytime dysfunction	↓ 0.50%^NS^	−0.02%^NS^
				Sleep efficiency (score)	↓ 1.45%^S^	−0.18%^S^
				Sleep quality	↑ 1.52%^S^	−0.34%^S^
				Sleep medication use	↓ 0.88%^NS^	0.07%^NS^
				Total score	↓ 8.20^S^	−1.20%^S^
				Epworth Sleepiness Scale	↓ 9.18%^S^	−1.52%^S^
33. Verma et al., 2023 (53)	RCT	*n*: 120 Age: 32.86 ± 7.08 Sex: *F* = 47, M = 73 Medical condition: insomnia	Type: integrated yoga Frequency (times per week): 6 Time (min): 60 Duration (weeks):8	PSQI score	↓ NR%^S^	NR%^S^
34. Harputlu et al., 2023 (54)	RCT	*n*: 55 Age: 56.67 ± 16.89 Sex: F = 23, M = 32 Medical condition: women with a history of fecal ostomies	Type: laughter yoga Frequency (times per week): 1 Time (min): 40–45 Duration (weeks): 8	PSQI score	↓ 6.16%^S^	0.52%^NS^
35. Metri et al., 2023 (55)	RCT	*n*: 38 Age: 39.37 ± 6.97 Sex: F Medical condition: chronic musculoskeletal pain	Type: integrated yoga Frequency (times per week): 4 Time (min): 60 Duration (weeks): 6	*(PSQI)*		
				Global score	↓ 4.80%^S^	−0.83%^S^
				Daytime dysfunction	↓ NR%^S^	NR
				Habitual sleep efficiency	↑ NR%^S^	NR
36. Uebelacker et al., 2023 (72)	RCT	*n*: 42 Age: 15.00 ± 1.50 Sex: *F* = 35, M = 7 Medical condition: depression	Type: integrated yoga Frequency (times per week): 1 Time (min): 45 Duration (weeks): 12	PROMIS sleep disturbance	↓ 24.23%^S^	1.13%^NR^
37. Jacoby et al., 2024 (56)	RCT	*n*: 226 Age: 33.37 ± NA Sex: *F* = 159, M = 67 Medical condition: generalized anxiety disorder	Type: Kundalini yoga Frequency (times per week): 1 for the supervised sessions and 7 for the home Time (min): 60 for the supervised sessions and 20 for the home Duration (weeks): 12	PSQI score	↓ 7.70%^S^	No CG
				Insomnia severity	↓ 11.32%^S^	
38. Dordevic et al., 2024 (86)	RCT	*n*: 173 Age: 53.33 ± NA Sex: F Medical condition: breast cancer	Type: NR Frequency (times per week): 2 Time (min): 45 Duration (weeks): 6	PSQI score	↓ NR%^NR^	NR%^S^
39. Ozmen and Unuvar., 2024 (57)	RCT	*n*: 90 Age: 35.60 ± 7.63 Sex: *F* = 41, M = 49 Medical condition: temporomandibular dysfunction	Type: face yoga Frequency (times per week): 3 Time (min): NR Duration (weeks): 6	PSQI score	↓ 10.38%^S^	1.98%^S^
40. Namdar et al., 2021 (58)	RCT	*n*: 60 Age: NR Sex: F Medical condition: low back pain	Type: hatha yoga Frequency (times per week): 2 Time (min): 75 Duration (weeks): 12	PSQI score	↓ 5.05%^S^	−2.64%^S^
41. Khalsa, 2004 (67)	Non-CS	*n*: 20 Age: 48.10 ± 10.00 Sex: *F* = 18, M = 2 Medical condition: insomnia	Type: Kundalini yoga Frequency (times per week): NR Time (min): 60 Duration (weeks): 8	Total wake time	↓ 2.22%^S^	No CG
				Total sleep time	↑ 5.72%^S^	
				Sleep efficiency (%)	↑ 0.71%^S^	
				Sleep quality	↑ 2.86%^NS^	
				Sleep onset latency	↓ NR%^S^	
				Number of awakenings	NR%^NS^	
				Wake time after sleep onset	↓ NR%^S^	
				Quality restedness at wake time	NR%^NS^	
42. Taibi and Vitiello, 2011 (34)	Non-CS	*n*: 13 Age: 65.20 ± 6.90 Sex: F Medical condition: osteoarthritis	Type: nidra yoga Frequency (times per week): 1 for the supervised classes and 7 for the home Time (min): 90 for the supervised classes and 20 for the home Duration (weeks): 8	Insomnia Severity Index	↓ 13.20%^S^	No CG
				Epworth Sleepiness Scale	↓ 7.55%^NS^	
				*(PSQI)*		
				Total score	↓ 9.10%^NS^	
				Daytime dysfunction	↓ 1.15%^S^	
				Sleep latency	↓ 1.23%^NS^	
				Habitual sleep efficiency	↑ 0.84%^NS^	
				Sleep quality	↓ 1.50%^S^	
				Sleep duration	NS	
				Sleep disturbance	NS	
				Sleep medication use	NS	
				*(Actigraphy)*		
				Sleep onset latency	NS	
				Sleep efficiency (%)	NS	
				Total sleep time	NS	
				Wake after sleep onset	NS	
				*(Sleep diaries)*		
				Sleep onset latency	↓ 0.37%^S^	
				Sleep efficiency (%)	↑ 0.79%^S^	
				Total sleep time	↑ 6.48%^NS^	
				Wake after sleep onset	NS	
				Number of nights with insomnia symptoms	↓ 3.90%^S^	
				Sleep quality	NS	
				Refreshment after sleeping	NS	
				Average daily joint pain	NS	
				Daytime sleepiness	NS	
43. Innes et al., 2013 (71)	Non-CS	*n*: 13 Age: 43.80 ± 4.21 Sex: F Medical condition: RLS	Type: Iyengar yoga Frequency (times per week): 2 for gym and 5 home Time (min): 90 for gym and 30 for home Duration (weeks): 8	*(Restless legs symptoms and severity)*		No CG
				IRLS symptom total	↓ 15.95%^S^	
				Symptom severity subscale	↓ 10.75%^S^	
				Symptom impact subscale	↓ 3.30%^NS^	
				RLS severity scale	↓ 3.25%^S^	
				*Sleep (Medical Outcomes Study Sleep Scale)*		
				Sleep problems index I	↓ 40.41%^S^	
				Sleep problems index II	↓ 44.16%^S^	
				Sleep disturbance	↓ 13.12%^S^	
				Snoring	↓ 16%^NS^	
				Sleep short of breath or headache	↓ 14%^NS^	
				Sleep adequacy scale	↑ 31%^S^	
				Somnolence scale	↓ 36.51%^S^	
				Total sleep duration	↑ 6.30%^NS^	
44. Halpern et al., 2014 (59)	Non-RCT	*n*: 90 Age: 73.48 ± 7.17 Sex: *F* = 74, M = 16 Medical condition: insomnia	Type: hatha yoga Frequency (times per week): 2 Time (min): NR Duration (weeks): 12	*(PSQI)*		
				Global score	↓ 9.24%^S^	−0.83%
				Sleep quality	↑ 1.43%^S^	−0.35%
				Sleep latency	↓ 1.67%^S^	−0.55%
				Sleep duration	↑ 1.88%^S^	−0.27%
				Sleep efficiency	↑ 1.23%^S^	−0.45% ^S^
				Sleep disturbance	↓ 1.30%^NS^	0.03% ^NS^
				Sleep medication	↓ 1.35%^NS^	−0.40% ^N^
				Sleep dysfunction	↓ 0.77%^NS^	−0.13% ^N^
				Sleep-wave-sleep duration	↑ 1.82%^NS^	0.03%^NS^
45. Milbury et al., 2015 (68)	Non-RCT	*n*: 20 Age: 69.99 ± 6.07 Sex: *F* = 14, M = 6 Medical condition: lung cancer	Type: couple-based Tibetan yoga Frequency (times per week): 2–3 Time (min): 45–60 Duration (weeks): 5–6	PSQI score	Patients: ↓ 11.76%^NS^ Caregivers: ↓ 10.62%^NS^	No CG
46. Milbury et al., 2015 (60)	Non-RCT	*n*: 30 Age: 60.55 ± 14.85 Sex: *F* = 10, M = 20 Medical condition: lung cancer	Type: Vivekananda yoga Frequency (times per week): 2–3 Time (min): 60 Duration (weeks): 5–6	PSQI score	Patients: ↓ 11.50%^NS^ Caregivers: ↓ 12.28%^S^	No CG
47. Buchanan et al., 2017 (35)	Non-CS	*n*: 17 Age: 54.58^†^ (50–72^‡^) Sex: *F* = 8, M = 9 Medical condition: insomnia	Type: viniyoga yoga Frequency (times per week): 1 Time (min): 90 Duration (weeks): 12	Insomnia Severity Index	↓ NR%^S†^	No CG
				Sleep disturbance	↓ NR%^S†^	
				Sleep-related impairment	↔^†^	
				*(Sleep diary)*		
				Time in bed	↔^†^	
				Total sleep time	↓ NR%^NS†^	
				Sleep latency	↑ NR%^NS†^	
				Wake after sleep onset	↑ NR%^NS†^	
				Sleep efficiency	↓ NR%^NS†^	
				Sleep quality	↑ NR%^NS†^	
				*(Actigraphy)*		
				Time in bed	↓ NR%^NS†^	
				Total sleep time	↑ NR%^NS†^	
				Sleep latency	↑ NR%^NS†^	
				Wake after sleep onset	↓ NR%^NS†^	
				Sleep efficiency	↓ NR%^NS†^	
48. Middleton et al., 2018 (73)	Non-CS	*n*: 30 Age: 49.50 ± NR Sex: *F* = 28, M = 2 Medical condition: arthritis	Type: hatha yoga Frequency (times per week): 2 Time (min): 60 Duration (weeks): 8	PROMIS sleep disturbance	↓ 49.40%^NS^	No CG
49. Chhugani et al., 2018 (65)	Non-RCT	*n*: 36 Age: 34.00 ± 8.40 Sex: F Medical condition: Alzheimer’s	Type: integrative yoga Frequency (times per week): 6 Time (min): 60 Duration (weeks): 4	PSQI score	↓ 9.07%^S^	−0.82%^S^
50. Daga et al., 2018 (61)	Non-CS	*n*: 100 Age: 18–61^‡^ Sex: *F* = 26, M = 74 Medical condition: sleep apnea	Type: pranayama and yoga asanas Frequency (times per week): 1 Time (min): 35 Duration (weeks): 24	PSQI score	↓ 12.37%^S^	No CG
				Epworth Sleepiness Scale	↓ 5.93^S^	
51. Lazaridou et al., 2019 (62)	Non-CS	*n*: 36 Age: 48.50 ± 13.90 Sex: F Medical condition: fibromyalgia	Type: Satyananda and home yoga Frequency (times per week): 6 (home) and 1 (Satyananda) Time (min): 30 (home) and 90 (Satyananda) Duration (weeks): 6	PSQI score	↓ 10.72%^S^	No CG
				Sleep efficiency	↑ 0.86%^NS^	
52. Kumar et al., 2019 (63)	Non-CS	*n*: 29 Age: 18–75^‡^ Sex: F = 8, M = 21 Medical condition: sleep apnea	Type: yogasana and traditional yoga Frequency (times per week): NR Time (min): NR Duration (weeks): 12	Epworth Sleepiness Score	↓ 10.77%^S^	No CG
				PSQI score	↓ NR%^S^	
				Snoring frequency	↓ NR%^S^	
				Snoring intensity	↓ NR%^S^	
				OSA severity	↓ NR%^S^	
53. Spadola et al., 2020 (70)	Non-CS	*n*: 17 Age: 43.60 ± 19.30 Sex: F = 15, M = 2 Medical condition: healthy	Type: Iyengar yoga Frequency (times per week): 4 Time (min): 60 Duration (weeks): 6	Sleep duration	↑ 6.14%^S^	No CG
				PROMIS Sleep-Related Impairment item	↓ 52.53%^S^	
				PROMIS Sleep Disturbance instruments	↓ 55.26%^S^	
				Sleep Hygiene Index	↑ 31.25%^S^	
54. Tunuguntla et al., 2021 (75)	Non-CS	*n*: 820 Age: 26–47^‡^ (68%), 48–58^‡^ (23%), 59–69^‡^ (8%), 18–25^‡^ (6.3%) Sex: *F* = 441, M = 378, prefer not to say = 1 Medical condition: insomnia	Type: app-based Yoga of Immortals Frequency (times per week): 2 Time (min): NR Duration (weeks): 8	*(Insomnia Severity Index)*		No CG
				Severe insomnia	↓ 21.83%^S^	
				Moderate insomnia	↓ 15.20%^S^	
				Subthreshold insomnia	↓ 9.23%^S^	
55. Gao et al., 2022 (69)	Non-CS	*n*: 89 Age: 19.88 ± 1.13 Sex: F Medical condition: healthy	Type: aromatherapy yoga and only yoga Frequency (times per week): 1 Time (min): 90 Duration (weeks): 12	*(PSQI)*		No CG
				Total score	AY: ↓ 6.76%^NS^ Y: ↑ 6.76%^NS^	
				Sleep quality	AY: ↑ 1.04%^NS^ Y: ↓ 0.99%^NS^	
				Sleep latency	AY: ↓ 1.39%^NS^ Y: ↓ 1.34%^NS^	
				Sleep duration	AY: ↓ 0.89%^NS^ Y: ↓ 1.01%^NS^	
				Habitual sleep efficiency	AY: ↓ 0.35%^NS^ Y: ↓ 0.28%^NS^	
				Sleep disturbance	AY: ↓ 1.19%^S^ Y: ↓ 1.19%^NS^	
				Use of sleep medication	AY: ↑ 0.04%^NS^ Y: ↔	
				Daytime dysfunction	AY: ↑ 1.83%^NS^ Y: ↑ 1.88%^NS^	
56. Turmel et al., 2022 (64)	Non-CS	*n*: 21 Age: 45 (28–58^‡^) Sex: *F* = 12, M = 9 Medical condition: insomnia	Type: viniyoga Frequency (times per week): 7 Time (min): 90 for the first week and 60 for the next 13 weeks Duration (weeks): 14	*(Polysomnography)*		No CG
				Total sleep time	↑ NR%^NS†^	
				Sleep efficiency	↑ NR%^NS†^	
				Sleep onset latency	↓ NR%^NS†^	
				REM latency	↑ NR%^NS†^	
				Stage N1	↓ NR%^NS†^	
				Stage N2	↓ NR%^NS†^	
				Stage N3	↑ NR%^NS†^	
				Stage REM	↓ NR%^NS†^	
				Arousal index	↓ NR%^NS†^	
				*(Actigraphy)*		
				Time in bed	↓ NR%^NS†^	
				Total sleep time	↑ NR%^NS†^	
				Sleep efficiency (%)	↑ NR%^NS†^	
				Arousals	↓ NR%^S†^	
				Naps	↑ NR%^NS†^	
				PSQI score	↓ NR%^S†^	
				Epworth Sleepiness Scale	↓ NR%^S†^	
57. Basavegowda et al., 2023 (74)	Non-CS	*n*: 173 Age: 25.92 ± 8.75 Sex: F Medical condition: insomnia	Type: yoga module Frequency (times per week): 6 Time (min): 60 Duration (weeks): 6	*(Insomnia severity index)*		No CG
				Subthreshold insomnia	↓ NR%^S†^	
				Moderate insomnia	↓ NR%^S†^	

n, number; F, female; M, male; ↑, an improvement in the outcome; ↓, a reduction in the outcome; ↔, no change; †, reported in median; ‡, reported in range; ¥, reported based on the change in participant numbers; S, significant; CG, control group; NS, not significant; NR, not reported; NA, not applicable; RCT, randomized controlled trial; Non-CS, non-controlled study; PSQI, Pittsburg Sleep Quality Index; PSAS, Pre-Sleep Arousal Scale; PROMIS, Patient Reported Outcomes Measurement Information System; RLS, restless legs syndrome; IRLS, International RLS Rating Scale; OSA, obstructive sleep apnea; AY, aromatherapy yoga; Y, yoga; QOL, quality of life.

### 3.3 Yoga characteristics

The studies included a diverse range of yoga types, such as Tibetan, Kundalini, Iyengar, awareness, restorative, Patanjali, silver, nidra, yogasana, pranayama, medical, hatha, integrated, couple-based, Tibetan, Vivekananda, viniyoga, Satyananda, home-based, traditional, app-based Yoga of Immortals, aromatherapy, module, tele, laughter, and face. The frequency of the yoga interventions averaged 2.98 ± 1.77 sessions per week, with each session lasting approximately 66.19 ± 17.51 min. The duration of the studies varied as they had a minimum duration of 4 weeks and a maximum duration of 24 weeks, resulting in an average intervention period of 10.51 ± 4.60 weeks.

### 3.4 Effects of yoga on sleep measures

The 57 studies included in this study encompassed a diverse range of populations, all of whom experienced sleep problems, alongside various medical conditions. These conditions included cancer, depression, arthritis, restless legs syndrome, stress, hot flushes, Alzheimer’s disease, dysfunctional uterine bleeding, fibromyalgia, low back pain, fecal ostomies, chronic musculoskeletal pain, generalized anxiety disorder, temporomandibular dysfunction, and type 2 diabetes. In addition, some individuals faced sleep issues without any specific underlying medical conditions.

**Figure 2:**
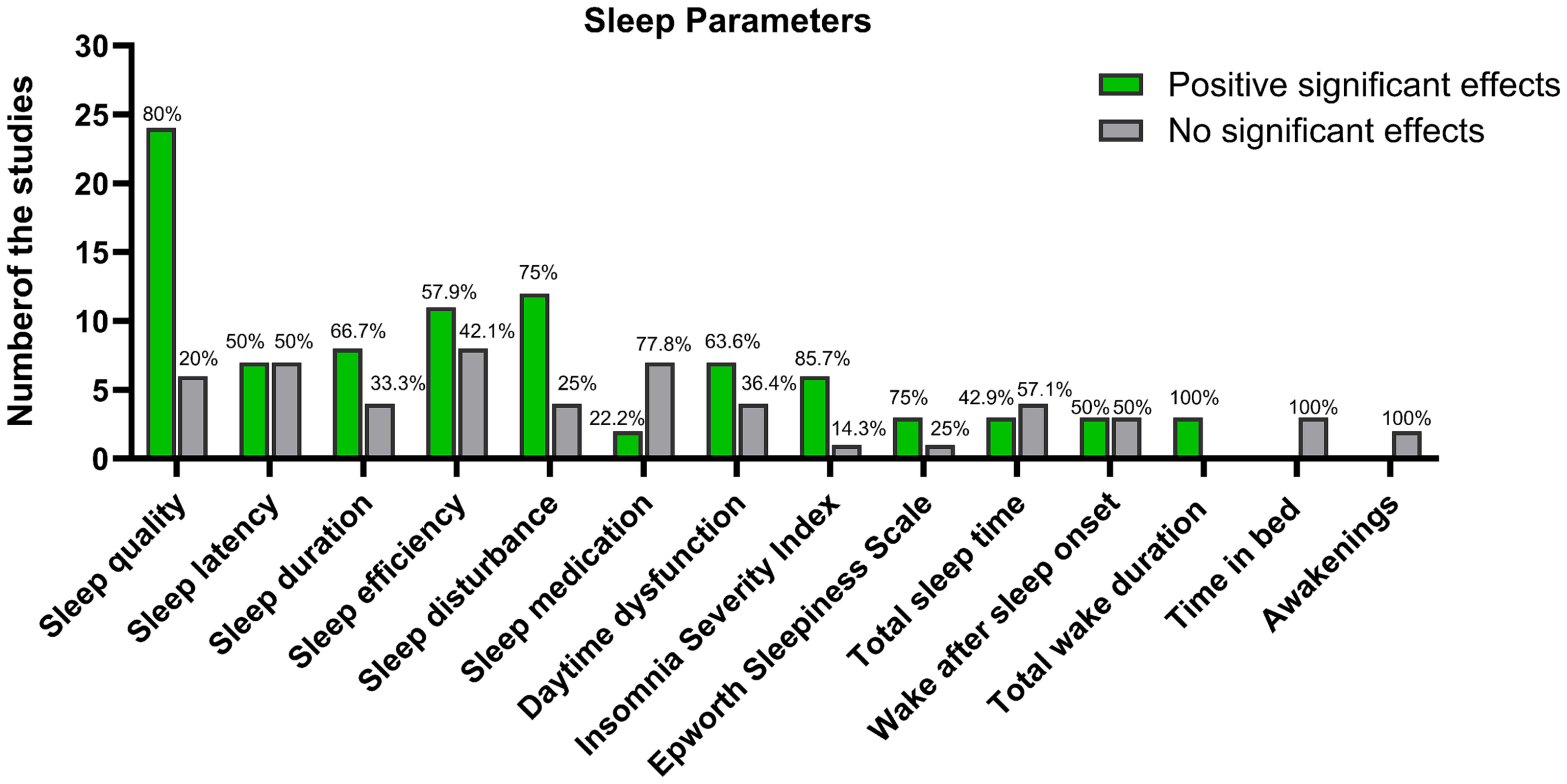
Summary of sleep parameters examined in the studies, categorized by their reported effects (positive significant effects or no significant effects).

The diverse characteristics of the yoga interventions yielded a complex range of outcomes concerning sleep quality across various measures comparing pre to post (Figure 2). Twenty-four studies reported statistically significant positive effects on sleep quality (42–65), while six studies found no significant effects (34, 60, 66–69). Regarding sleep latency, eight studies demonstrated significant positive effects (34, 44, 45, 48–50, 59, 67), whereas six studies showed no significant effects (34, 35, 46, 52, 64, 69). For sleep duration, seven studies reported statistically significant positive effects (43–45, 49, 52, 59, 70), while five studies showed no significant effects (34, 42, 46, 52, 69). Concerning sleep efficiency, 11 studies observed significant positive improvements (34, 43–45, 48–50, 52, 55, 59, 67), whereas eight reported no significant effects (34, 35, 42, 44, 46, 62, 64, 69). Regarding sleep disturbance, 12 studies indicated significant positive effects (35, 42–46, 49, 52, 69–72), while four studies found no significant effects (34, 59, 69, 73). For sleep medication usage, two studies reported significant positive effects, whereas seven studies showed no significant effects (34, 43–46, 49, 52, 59, 69). In terms of daytime dysfunction, seven studies demonstrated significant positive effects (34, 43–45, 49, 55), while four studies found no significant effects (46, 52, 59, 69). Regarding the Insomnia Severity Index, six studies reported positive effects (34, 35, 56, 74–76), whereas one study found no significant effects (50). For the Epworth Sleepiness Scale, three studies revealed positive effects, and one study showed no significant change (34, 52, 61, 64). Concerning total sleep time and wake after sleep onset, three studies demonstrated positive effects, while seven studies showed no significant effects (34, 35, 44, 48, 50, 64, 67, 71). Moreover, three studies found significant positive effects on total wake duration (48, 50, 67). Finally, three studies and two studies reported non-significant effects for time in bed and awakenings, respectively (35, 48, 50, 64, 67).

### 3.5 Moderating variables in yoga intervention studies on sleep outcomes

#### 3.5.1 Duration of yoga interventions

Duration of yoga interventions plays a crucial role in determining the effectiveness of yoga on sleep outcomes.

##### 3.5.1.1 Short duration (≤6 weeks)

Approximately 54% of 13 studies in total with short durations showed statistically significant improvement in sleep measures (44, 55, 57, 62, 65, 70, 74). The mean effect of the yoga intervention on sleep quality demonstrated a large mean effect of 9.41% (95% CI 3.06 to 15.42%), based on data from seven studies. Of these, five reported significant improvements, while two studies showed no significant change.

##### 3.5.1.2 Medium duration (7–16 weeks)

This category comprised approximately 57% of the 40 studies reporting statistically significant improvements across sleep measures (34, 35, 43, 45–47, 49, 50, 52–54, 56, 58, 59, 63, 64, 67, 69, 71, 72, 75–77). For sleep quality, there was a large mean effect of 8.74% (95% CI 2.93 to 14.55%), based on data synthesized from 14 studies. Of these, 11 studies showed significant improvements, while three reported no significant effects. Sleep efficiency exhibited a small mean effect of 0.73% (95% CI −1.99 to 3.45%), derived from data across eight studies. Among these, six studies indicated significant effects, while two studies found no significant change. Sleep disturbance demonstrated a large mean effect of 5.61% (95% CI 3.36 to 7.86%), based on findings from nine studies. Of these, seven indicated significant reductions, whereas two showed no significant effects. Sleep duration increased by 1.96% (95% CI 1.23 to 2.69%), reflecting a small effect based on data from seven studies. Of these, four studies reported significant improvements, while three indicated no significant change. For insomnia severity, there was a very large mean improvement of 13.19% (95% CI 11.10 to 15.98%), based on data from five studies. Among these studies, four demonstrated significant improvements, while only one reported no significant effect. Daytime dysfunction decreased by 1.06% (95% CI 0.55 to 1.57%), indicating a small effect, according to seven studies. Among these, three studies showed significant reductions, while four reported no significant effects. For the Epworth Sleepiness Scale, a large mean effect of 8.36% (95% CI 5.59 to 11.12) was derived from two studies. Of these, one study demonstrated a significant improvement, whereas the other showed no significant effect. For total sleep time, a large mean effect of 6.52% (95% CI 5.71 to 7.32%) was identified from three studies. Of these, one study showed a significant increase, while two studies reported no significant change. Moreover, six studies showed a small mean effect of 0.56% (95% CI −1.62 to 2.74%) with regard to the use of sleep medication. Of these, one study showed a significant reduction, while five studies demonstrated no significant effects.

##### 3.5.1.3 Long duration (≥17 weeks)

The long-duration group yielded the most substantial results, with 100% of a total of three studies showing statistically significant improvements in various sleep outcomes, including sleep efficiency, sleep quality, and sleep disturbance reduction (42, 51, 61). For sleep quality, there was a large improvement of 7.92% (95% CI 3.23 to 12.60%), as reported in the data from the three studies.

#### 3.5.2 Frequency of yoga sessions per week

The frequency of yoga practice moderates its impact on sleep outcomes:

##### 3.5.2.1 Low frequency (1–2 sessions per week)

Research in this area revealed significant enhancements in sleep quality, with a large mean effect of 8.13% (95% CI 2.67 to 13.59%) derived from nine studies. Of these, seven studies reported significant improvements, while two found no significant effects. Sleep disturbances demonstrated a moderate reduction, as indicated by a mean effect of 3.30% (95% CI 1.34 to 5.26%) from six studies. Among these, four studies reported significant reductions, while two studies showed no significant effects. Sleep duration improved with a small magnitude, yielding a mean effect of 1.28% (95% CI –0.51 to 3.07%) from three studies. Sleep efficiency also showed a small effect, with a mean change of 0.93% (95% CI: −10.69 to 12.55%) reported across five studies. Among these, three studies exhibited significant improvements, while two studies showed no significant change. Sleep latency experienced a small decrease, with a mean effect of 1.35% (95% CI −1.07 to 3.77%) from four studies. Of these, two studies reported significant reductions, while the other two showed no significant effects. Furthermore, daytime dysfunction was reduced, reflecting a small mean effect of 1.29% (95% CI −0.55 to 3.13%) from four studies. Among these, two studies indicated significant reductions, while the other two found no significant effects. Lastly, insomnia severity decreased significantly, with a very large mean effect of 13.66% (95% CI 8.72 to 18.59%) from two studies. Moreover, with regard to the use of sleep medication, there was a small mean effect of 0.58% (95% CI −1.94 to 3.10%) from four studies.

##### 3.5.2.2 Moderate frequency (3–4 sessions per week)

This frequency level resulted in a large mean effect on sleep quality of 9.21% (95% CI 3.66 to 14.76), based on data from 10 studies. Of these, eight studies showed significant improvements, while two studies reported no significant change. For sleep duration, a moderate mean effect of 2.99% (95% CI 0.45 to 5.53%) was observed, based on five studies. Among these, three studies reported significant improvements, while two showed no significant change. For sleep latency, a small mean effect of 1.06% (95% CI −1.45 to 3.57%) was identified in three studies. Among these, one study reported a significant reduction, while two studies showed no significant effects. Sleep disturbances exhibited a moderate mean effect of 2.35% (95% CI 0.58 to 4.12%), based on five studies. Of these, three studies reported significant reductions, while two showed no significant effects. For sleep efficiency, a small mean effect of 0.61% (95% CI −1.52 to 2.74%) was observed, based on data from four studies. Among these, two studies indicated significant improvements, while the other two found no significant effects. For daytime dysfunction, a small mean effect of 0.60% (95% CI −1.42 to 2.62%) was identified from the data synthesized from four studies. Of these, two studies reported significant reductions, while the other two showed no significant effects. In addition, a small mean effect of 0.65% (95% CI − .18 to 2.48%) was reported with regard to sleep medication, as reported from the data of three studies. One study showed a significant reduction, while two studies reported no significant effects.

##### 3.5.2.3 High frequency (≥5 sessions per week)

Participants practicing yoga at this intensity experienced a large mean effect on sleep quality of 8.24% (95% CI 2.28 to 14.20%), based on data from three studies. In addition, for sleep efficiency, a small mean effect of 0.84% (95% CI −3.27 to 4.95) was observed, aggregated from two studies. Of these, one study showed a significant improvement, while the other reported no significant effect.

## 4 Discussion

This scoping review primarily aims to examine the impact of chronic yoga interventions on sleep quality among individuals experiencing sleep problem syndrome. The synthesis of the findings reveals a connection between the various types of yoga and improvements in sleep quality. The results are bolstered by research encompassing diverse populations with varying medical conditions and age groups. Yoga has demonstrated significant positive effects on various aspects of sleep quality, including sleep latency, duration, efficiency, and disturbance. It enhances total sleep time while reducing wake after sleep onset and total wake duration, leading to improved overall sleep satisfaction. In addition, yoga decreases the need for sleep medication and alleviates daytime dysfunction. Assessments such as the Insomnia Severity Index and the Epworth Sleepiness Scale further highlight improvements in insomnia symptoms and daytime sleepiness, showcasing yoga’s comprehensive benefits for sleep health.

This scoping review aligns with existing literature on non-pharmacological interventions for sleep disorders, particularly regarding the effectiveness of yoga. Gao et al. (69) reported that yoga significantly improved sleep disorders, sleep efficiency, and sleep duration, corroborating our findings that yoga enhances sleep quality and latency. Similarly, Alnawwar et al. (78) emphasized the role of regular physical activity, including moderate-intensity exercises such as yoga, in improving overall sleep quality and reducing sleep latency. However, Alimoradi et al. (31) noted that not all modalities, such as stretch training, show significant improvements, highlighting variability in outcomes across different populations and intervention designs. While yoga has demonstrated substantial benefits, it is crucial to compare its effectiveness with other non-pharmacological interventions. For example, Chen et al. (79) found that Pilates significantly improved sleep quality, as evidenced by reductions in PSQI scores, suggesting that it may be as effective as yoga for enhancing sleep among various populations. Cognitive and behavioral interventions, such as cognitive behavioral therapy for insomnia (CBT-I), have also shown robust benefits for sleep health (80). CBT-I remains the gold standard for sleep disorder treatment due to its ability to address maladaptive sleep-related thoughts and behaviors (81). However, unlike CBT-I, yoga provides additional physiological benefits, such as autonomic nervous system regulation (43), stress hormone reduction (74), and improved cardiovascular function (82), which may contribute to its effectiveness in improving sleep. In the context of exercise-based interventions, Yang et al. (83) demonstrated that moderate-intensity aerobic and high-intensity resistance exercises improve sleep quality, as indicated by better PSQI scores. However, resistance training appears less effective when combined with aerobic exercise, potentially diminishing its benefits compared to aerobic activity alone (84). Moreover, while resistance exercise has shown promise, its acute effects on sleep remain inconsistent (84). Given that mobility restrictions or chronic pain may limit participation in high-impact exercise programs, yoga could serve as a more accessible alternative for individuals with such conditions.

In summary, while this review highlights yoga as an effective intervention for improving sleep quality, it is clear that other modalities, such as Pilates, cognitive and behavioral therapies, moderate-intensity aerobic exercise, and resistance training, also provide significant benefits. Future research should conduct direct comparisons between yoga and these interventions to determine its relative efficacy and suitability for different populations. Additionally, further studies should explore the optimal conditions for these interventions and examine factors influencing individual responses to different types of exercise for sleep improvement.

### 4.1 The role of duration and frequency in sleep outcomes

The duration of the yoga interventions significantly influences their effectiveness on sleep outcomes, highlighting the need for careful consideration in therapeutic applications. Short-duration interventions (≤6 weeks) show some efficacy, but their impact is limited, with only about half of the studies reporting significant improvements (44, 55, 57, 62, 65, 70, 74). This raises questions about the sustainability of benefits from brief practices and suggests that longer interventions may be necessary to achieve more profound effects. Medium-duration interventions (7–16 weeks) demonstrate a broader range of positive outcomes, indicating that this duration may represent an optimal balance between commitment and effectiveness (34, 35, 43, 45–47, 49, 50, 52–54, 56, 58, 59, 63, 64, 67, 69, 71, 72, 75–77). However, the most compelling evidence emerges from the long-duration interventions (≥17 weeks), which consistently yield significant improvements across various sleep metrics (42, 51, 61). This pattern indicates that longer practices can enhance sleep quality and could potentially lead to lasting changes in sleep health, suggesting a possible dose–response relationship. Therefore, future research should prioritize exploring the mechanisms by which duration influences outcomes, as well as the potential for tailored intervention lengths, to maximize benefits for individuals experiencing sleep disturbances.

The frequency of the yoga sessions plays a pivotal role in shaping the impact of yoga on sleep outcomes. Low-frequency practices (1–2 sessions per week) demonstrate significant improvements in sleep quality and insomnia severity, suggesting that even minimal engagement can yield beneficial effects (43, 44, 47, 54, 58, 59, 61, 69, 71, 75, 76). However, the relatively modest improvements in the other sleep metrics raise questions about the sufficiency of this frequency for comprehensive sleep enhancement. Moderate-frequency sessions (3–4 times per week) appear to produce more pronounced benefits, indicating that increased engagement may enhance the therapeutic effects of yoga on sleep disturbances and duration (42, 45, 46, 49, 51, 52, 55, 57, 70). Interestingly, high-frequency practices (≥5 sessions per week) also show positive outcomes, but the diminishing returns observed suggest that there may be an optimal frequency for maximizing benefits without leading to fatigue or burnout (50, 62, 65). Overall, these findings underscore the importance of tailoring yoga interventions to individual preferences and needs, as the frequency of practice can significantly influence the effectiveness of yoga in improving sleep health. Future research should explore the mechanisms underlying these frequency effects and consider how individual variability may impact responsiveness to different practice schedules.

### 4.2 Exploring the mechanisms of yoga in potentially enhancing sleep quality

**Figure 3:**
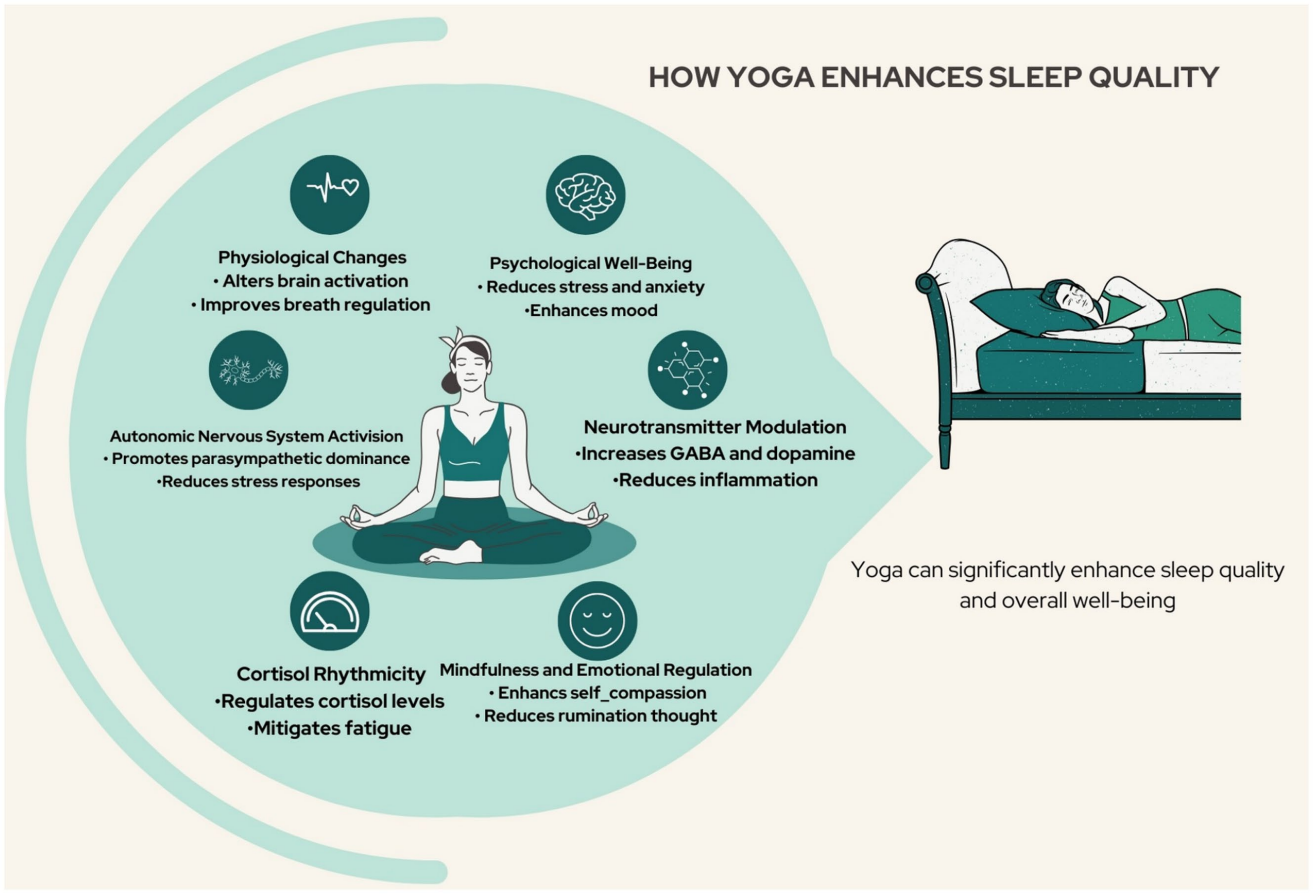
Potential mechanisms enhancing sleep quality for individuals with sleep problems.

The relationship between yoga and sleep quality is complex, involving several interrelated mechanisms that contribute to improved sleep among individuals facing sleep difficulties (Figure 3).

#### 4.2.1 Psychological well-being and stress reduction

Regular yoga practice has been shown to reduce stress levels and enhance psychological well-being, both of which are crucial for promoting better sleep (74). By fostering a sense of relaxation and acceptance, yoga helps alleviate anxiety and depression, which are common contributors to sleep disturbances (77).

#### 4.2.2 Physiological changes

Methodological advancements in research have indicated that yoga can lead to physiological changes, such as alterations in anterior insular cortex activation. These changes, coupled with practices such as breathwork and mindfulness, have demonstrated efficacy in reducing anxiety and enhancing sleep quality (85). Specifically, pranayama or yogic breathing techniques can interact with the nervous system to influence metabolic and autonomic functions. Jerath et al. (48) suggested that slow, deep breathing can reset the autonomic nervous system, promoting parasympathetic dominance associated with improved sleep quality.

#### 4.2.3 Impact on neurotransmitters and inflammation

Emerging evidence suggests that yoga modulates various physiological aspects, including neurotransmitter levels and inflammation markers. Reductions in pro-inflammatory markers alongside increased gamma-aminobutyric acid (GABA) levels indicate a regulatory effect on mood and well-being through inflammation control (86). Neuroimaging studies have shown that yoga selectively activates neurochemical systems involved in sleep regulation, increasing dopamine and GABA levels, which are crucial for pain processing (87).

#### 4.2.4 Mindfulness and emotional regulation

Improvements in mindfulness cultivated through yoga can enhance emotional regulation while decreasing hyperarousal and ruminative thoughts, which are factors known to disrupt sleep (88). In addition, the promotion of self-compassion and a mindful perspective through yoga can further mitigate stress and anxiety, thereby enhancing cognitive function and overall quality of life (72).

#### 4.2.5 Autonomic nervous system activation

Yoga promotes relaxation by reducing sympathetic nervous system activation and hypothalamic–pituitary–adrenal (HPA) axis reactivity, which have both been implicated in sleep disturbances (43). This relaxation response not only alleviates pain associated with sleep issues but also improves overall well-being. Furthermore, yoga’s ability to enhance parasympathetic output through vagus nerve stimulation contributes to better sleep and improved mood (50).

#### 4.2.6 Cortisol rhythmicity

Alterations in cortisol rhythmicity linked to yoga practice can also impact behavioral symptoms such as fatigue and depression (89).

Collectively, these mechanisms underscore the potential of yoga as a non-pharmacological intervention for improving sleep quality across diverse populations experiencing sleep disturbances.

In summary, the multifaceted mechanisms through which yoga enhances sleep quality highlight its potential as an effective non-pharmacological intervention for those struggling with sleep issues.

### 4.3 Consideration of comorbidities in sleep disturbances

It is important to acknowledge the potential influence of comorbidities, such as headaches and psychiatric disorders, on sleep disturbances. While this scoping review focused primarily on the effects of chronic yoga interventions on sleep quality, it is well-established that comorbid conditions can both exacerbate and be exacerbated by sleep problems. For instance, chronic headaches, including migraines, have been shown to significantly impact sleep patterns, potentially contributing to increased sleep disturbances (90). In turn, poor sleep quality may aggravate headache severity, creating a vicious cycle of discomfort and disrupted sleep.

Similarly, psychiatric disorders such as anxiety, depression, and insomnia have a bidirectional relationship with sleep disturbances. These conditions often co-occur, and the presence of mental health conditions can significantly alter sleep architecture, leading to issues such as insomnia, poor sleep efficiency, and prolonged sleep onset latency. This interaction is especially relevant in the context of yoga interventions, as yoga has been demonstrated to alleviate both psychological symptoms and sleep disturbances (91). While the current review synthesizes evidence on yoga’s positive effects on sleep, future research should consider the presence of such comorbidities to better understand the complex relationship between mental and physical health conditions and sleep quality. Including comorbid conditions in future studies may help elucidate whether the observed benefits of yoga on sleep are universally applicable or specific to certain subsets of individuals, especially those with concurrent health issues.

### 4.4 Strengths and limitations

The strengths of this scoping review are underscored by its rigorous adherence to the PRISMA Scoping Review Checklist (38), which ensures a robust methodology throughout both the conduct and reporting phases. The comprehensive search protocol employed major medical research databases, citation searching, and efforts to identify unpublished studies, thereby enhancing the inclusion of high-quality research. With a total of 57 studies reviewed, the breadth of data allows for a more nuanced understanding of the effects of chronic yoga interventions on sleep quality across various populations experiencing sleep problem syndrome. However, several limitations must be acknowledged. A significant limitation of this scoping review is that the included studies did not uniformly use polysomnography (PSG), the gold standard for objectively diagnosing sleep disorders (92). This reliance on subjective measures of sleep quality, such as self-reported sleep diaries and questionnaires, may introduce bias or inaccuracies in the results. While these tools are widely used in sleep research, they do not provide the same level of precision as PSG. Furthermore, the considerable heterogeneity among the included studies, including variations in the types of yoga interventions, session frequency, and duration, complicates the interpretation of the results. In addition, inconsistencies related to diverse medical conditions may limit the generalizability of the findings. These factors, combined with the lack of a control group in the studies reviewed, highlight the need for caution when drawing definitive conclusions about the efficacy of yoga interventions for improving sleep quality in clinical contexts.

### 4.5 Future research directions

While this review highlights the promising role of yoga in sleep improvement, several gaps remain:

- Long-term follow-up studies are needed to determine whether the benefits of yoga persist after discontinuation.
- Direct comparative studies between yoga and other sleep interventions (e.g., CBT-I, aerobic exercise, pharmacotherapy) would clarify its relative effectiveness.
- Objective sleep assessments using actigraphy and polysomnography should be incorporated into future trials to provide more rigorous evidence.
- Population-specific investigations are needed to determine which groups (e.g., older adults, individuals with insomnia, those with chronic pain) benefit most from yoga-based interventions.

## 5 Conclusion

Chronic yoga interventions have been shown to significantly enhance sleep quality among diverse populations suffering from sleep problem syndrome. These interventions positively influence various sleep measures, including sleep latency, duration, and efficiency. The evidence suggests that yoga improves sleep quality through multiple mechanisms: it effectively reduces stress and anxiety, promotes physiological relaxation, and fosters mindfulness. Despite these encouraging results, there remains a pressing need for further investigation into yoga as a viable therapeutic option. Future studies should prioritize validating these interventions through larger-scale RCTs and examining the long-term benefits of chronic yoga practice on sleep quality. As the demand for non-pharmacological solutions to sleep disturbances grows, establishing a robust evidence base for yoga could facilitate its integration into mainstream therapeutic practices for individuals affected by sleep problem syndrome.

## Data Availability

The datasets presented in this study can be found in online repositories. The names of the repository/repositories and accession number(s) can be found in the article.

## Footnotes

^1^ https://www.connectedpapers.com/
